# Pathways of Amino Acid Degradation in *Nilaparvata lugens* (Stål) with Special Reference to Lysine-Ketoglutarate Reductase/Saccharopine Dehydrogenase (LKR/SDH)

**DOI:** 10.1371/journal.pone.0127789

**Published:** 2015-05-22

**Authors:** Pin-Jun Wan, San-Yue Yuan, Yao-Hua Tang, Kai-Long Li, Lu Yang, Qiang Fu, Guo-Qing Li

**Affiliations:** 1 State Key Laboratory of Rice Biology, China National Rice Research Institute, Hangzhou 310006, China; 2 Education Ministry Key Laboratory of Integrated Management of Crop Diseases and Pests, College of Plant Protection, Nanjing Agricultural University, Nanjing 210095, China; Zhejiang University, CHINA

## Abstract

*Nilaparvata lugens* harbors yeast-like symbionts (YLSs). In present paper, a genome-wide analysis found 115 genes from *Ni*. *lugens* and 90 genes from YLSs that were involved in the metabolic degradation of 20 proteinogenic amino acids. These 205 genes encoded for 77 enzymes. Accordingly, the degradation pathways for the 20 amino acids were manually constructed. It is postulated that *Ni*. *lugens* can independently degrade fourteen amino acids (threonine, alanine, glycine, serine, aspartate, asparagine, phenylalanine, tyrosine, glutamate, glutamine, proline, histidine, leucine and lysine). *Ni*. *lugens* and YLSs enzymes may work collaboratively to break down tryptophan, cysteine, arginine, isoleucine, methionine and valine. We cloned a *lysine-ketoglutarate reductase/saccharopine dehydrogenase* gene (*Nllkr/sdh*) that encoded a bifunctional enzyme catalyzing the first two steps of lysine catabolism. *Nllkr/sdh* is widely expressed in the first through fifth instar nymphs and adults, and is highly expressed in the fat body, ovary and gut in adults. Ingestion of ds*Nllkr/sdh* by nymphs successfully knocked down the target gene, and caused nymphal/adult mortality, shortened nymphal development stage and reduced adult fresh weight. Moreover, *Nllkr/sdh* knockdown resulted in three defects: wings were shortened and thickened; cuticles were stretched and thinned; and old nymphal cuticles remained on the tips of legs and abdomen and were not completely shed. These data indicate that impaired lysine degradation negatively affects the survival and development of *Ni*. *lugens*.

## Introduction

In animals, amino acid degradation is critically important. Firstly, amino acids are one of the three major energy sources for animals. Amino acids as an energy source are especially important for carnivorous animals and for all animals during starvation. For example, in the brown planthopper *Nilaparvata lugens*, upregulated transcription level of a proline-degraded gene was observed due to starvation induced by the antifeedant activity of triazophos, and proline as an alternative energy source was then catabolized [1]. Lys-ketoglutarate reductase/saccharopine dehydrogenase (LKR/SDH) is a bifunctional enzyme catalyzing the first two degradation steps of lysine (Lys) in plants and animals. In a tick *Haemaphysalis longicornis*, the *Hllkr/sdh* transcripts were more abundant in starved individuals than in well-fed and engorged ones [2]. Moreover, an 82% increase in LKR/SDH mRNA and a 52% increase in LKR activity were observed in mice starved for 1–2 days [3]. All those findings suggest that more Lys is degraded in starved animals.

Secondly, herbivores tend to obtain free amino acids from plants. However, many plants contain low levels of some essential amino acids. For example, rice phloem sap, as the food of *Ni*. *lugens*, contains high levels of simple sugars but low levels of nitrogenous organic compounds such as free amino acids. Among those free amino acids in rice phloem saps, only asparagine (Asn), glutamate (Glu), glutamine (Gln), threonine (Thr) and valine (Val) are abundant [4]. Moreover, ingested free amino acids cannot be stored by *Ni*. *lugens*. The insect must degrade the excess amino acids that are not needed for protein synthesis to maintain a balanced amino acid composition in hemolymph.

Thirdly, an accumulation of amino acids and/or their breakdown intermediates is harmful to animals. For example, high levels of Lys were toxic to plant and mammalian cells [5–7]. In *Ha*. *longicornis*, silencing *Hllkr/sdh* by RNA interference (RNAi) seriously affected the osmotic regulation of water balance and egg development of engorged females [2]. In humans, defects that lead to accumulation of certain amino acids can cause severe illness [8–13].

*Ni*. *lugens* is a serious pest in paddy fields throughout Asia [14]. It harbors several species of yeast-like symbionts (YLSs) [15–17], mainly in abdominal fat bodies. Previously, according to a transcriptome deposited in NCBI (Accession No. SRX326774), we had manually constructed biosynthesis pathways for the 20 protein amino acids. We postulated that both *Ni*. *lugens* and its symbionts can independently biosynthesize seven non-essential amino acids of Glu, Gln, aspartate (Asp), Asn, alanine (Ala), serine (Ser) and glycine (Gly). *Ni*. *lugens* and symbiont enzymes may work collaboratively to catalyze the biosynthesis of proline (Pro), methionine (Met), Val, leucine (Leu), isoleucine (Ile), phenylalanine (Phe) and tyrosine (Tyr). And the symbionts alone may function in the biosynthesis of Lys, arginine (Arg), tryptophan (Trp), Thr, histidine(His) and cysteine (Cys) [18]. An interesting question is: Do planthoppers degrade amino acids for themselves?

The genomes of *Ni*. *lugens* and YLS were released recently [19]. They are useful in identification of genes involved in amino acid degradation. A genome-wide analysis allowed us to construct pathways for metabolic degradation of the 20 protein amino acids. More importantly, we cloned *Nllkr/sdh*, and showed that knocking down of this gene using double stranded-RNA (dsRNA) caused obvious negative effects. Our results indicate that degradation of Lys is critical for the survival and development of *Ni*. *lugens*.

## Materials and Methods

### Insect rearing

*Ni*. *lugens* were maintained on rice variety Taichung Native 1 (TN1) for more than 170 generations under controlled temperature (28 ± 1°C), relative humidity (80±10%) and photoperiod (14/10 h light/dark) in China National Rice Research Institute. TN1 seedlings were grown in soil at 30–35°C under a long day photoperiod (14/10 h light/dark) in a growth incubator. Planthoppers were transferred to fresh seedlings every 10–14 days to assure sufficient nutrition. All animal works were conducted according to relevant nation and international guidelines.

### Bioinformatics analysis

The annotated proteins involved in amino acid degradations from model insects *Drosophila melanogaster*, *Bombyx mori*, *Anopheles gambiae*, *Tribolium castaneum*, *Apis mellifera* and *Acyrthosiphon pisum*, and from model fungi *Schizosaccharomyces pombe*, *Saccharomyces cerevisiae* and *Metharhizium robertsii* were downloaded from the NCBI. These protein sequences were used for TBLASTN searches of *Ni*. *lugens* genome and YLS genome [19], respectively, to locate DNA hits. E-value was set at 10 in order to detect all possible genomic hits. Each genomic hit was extended by approximately 5,000 bp upstream and downstream to ensure coverage of the full-length of genes. The extended DNA sequences were then downloaded. Genes within the downloaded sequences were predicted by GenScan [20], augustus [21], FGENESH [22] and exonerate [23]. The predicted protein sequences of the genes were blasted (BLASTP, e-values <0.001) against NCBI non-redundant proteins (nr) to identify the highest hit sequences, which were then used as queries in exonerate analyses to extend the nucleotide sequences. Sequences were extended to their start and stop codons. Genes containing premature stop codons or frameshifts within the translation predicted by the exonerate analyses were considered as pseudogenes and removed.

In order to get transcriptional evidence of the genes, each gene was searched against a *de novo* transcriptome assembly. The transcriptome was assembled from the clean reads (accession no. SRX326774) using TRINITY [24]. Potential alternatively spliced expressed sequence tags (ESTs) or potential paralogous ESTs that shared common subsequences were also predicted using TRINITY.ESTs that exhibited perfect identity with the predicted genes were retained. The resulting genes and ESTs were annotated by the blastx applications in Blast2GO software [25]. The annotations were individually inspected.

### Identification of genes of host or YLS origin

The identified *Ni*. *lugens* and YLS genes were searched (blastx) against nr databases, respectively. All YLS genes shared the highest identities with those of *Metarhizium* spp, a closest relative to YLSs [19]. Among the *Ni*. *lugens* genes, sequences that had the greatest identity with that from *Rhodnius prolixus* or *Ac*. *pisum*, which are closely related to *Ni*. *lugens*, were considered as *Ni*. *lugens* (host) origin. The output from the blastx searches against nr databases for each gene was further analyzed with MEGAN v7.7.1 [26]. After exclusion of duplicate sequences, MEGAN assigned them to one of the nodes within insect or fungi clade in the NCBI taxonomy. Additionally, we found that some *Ni*. *lugens* sequences exhibited essentially perfect identity with *Arsenophonus nilaparvatae* genome signaling that they were either DNA contaminants or the results of very recent horizontal gene transfer (HGT) from bacteria. Those sequences were excluded from further analysis because it is beyond the scope of the present study. After manual filtering, the analysis produced a list of genes that encode enzymes involved in amino acid degradation (Table 1).

**Table 1:** Counts of identified genes from *Ni*. *lugens* and YLS genome.

EC number	Name	No. of genes from *Ni*. *lugens* genome^a^	No. of genes from YLS genome^a^
***Pyruvate degradation family (alanine*, *serine*, *glycine*, *cysteine*, *tyrosine*, *trytophan*, *phenylpyuvate*, *threonine)***			
1.13.11.11	Tryptophan 2,3-dioxygenase	1	1
3.5.1.9	Arylformamidase	1	1
1.14.13.9	Kynurenine 3-monooxygenase	1	1
3.7.1.3	Kynureninase	0	1
2.6.1.2	Alanine transaminase	4	1
2.6.1.44	Alanine—glyoxylate transaminase	2	1
2.6.1.45	Serine—glyoxylate transaminase	0	
4.1.2.5	L-threonine aldolase	2	2
2.1.2.1	Glycine hydroxymethyltransferase	1	2
4.3.1.19	Threonine ammonia-lyase	2	1
1.1.1.272	(R)-2-hydroxyacid dehydrogenase	2	5
2.6.1.1	Aspartate transaminase	1	2
4.3.1.17	L-serine ammonia-lyase	0	1
4.4.1.10	Cysteine lyase	0	1
4.4.1.25	L-cysteate sulfo-lyase	0	0
4.4.1.24	(2R)-sulfolactate sulfo-lyase	0	0
***Oxaloacetate and fumarate degradation family (aspartic acid*, *asparagine*, *tyrosine)***			
1.4.3.1	D-aspartate oxidase	2	2
5.1.1.13	Aspartate racemase	0	0
3.5.1.1	Asparaginase	2	1
6.3.4.4	Adenylosuccinate synthase	1	1
6.3.4.5	Argininosuccinate synthase	0	1
4.3.2.2	Adenylosuccinate lyase	0	1
4.3.2.1	Argininosuccinate lyase	0	1
3.7.1.2	Fumarylacetoacetase	1	2
5.2.1.2	Maleylacetoacetate isomerase	1	1
1.13.11.5	Homogentisate 1,2-dioxygenase	1	1
1.13.11.27	4-hydroxyphenylpyruvate dioxygenase	1	1
2.6.1.5	Tyrosine transaminase	3	1
1.14.16.1	Phenylalanine 4-monooxygenase	3	0
***α-ketoglutarate degradation family (glutamate*, *glutamine*, *proline*, *arginine*, *histidine*, *aspartate*, *alanine*, *tryptophan)***			
3.5.3.1	Arginase	0	3
2.6.1.13	Ornithine aminotransferase	3	0
1.5.1.12	1-pyrroline-5-carboxylate dehydrogenase	1	1
1.5.99.8	Proline dehydrogenase	1	1
1.4.1.2	Glutamate dehydrogenase	2	1
1.4.1.3	Glutamate dehydrogenase (NAD(P)(+))		
1.4.1.4	Glutamate dehydrogenase (NADP(+))		
4.3.1.3	Histidine ammonia-lyase	1	3
4.2.1.49	Urocanate hydratase	1	0
3.5.2.7	Imidazolonepropionase	1	1
2.1.2.5	Glutamate formimidoyltransferase	2	0
3.5.1.2	Glutaminase	1	0
2.6.1.16	Glutamine-fructose-6-phosphate transaminase (isomerizing)	1	1
2.4.2.14	Amidophosphoribosyltransferase	3	1
6.3.5.5	Carbamoyl-phosphate synthase (glutamine-hydrolysing)	2	4
***Succinyl-CoA degradation family (valine*, *isoleucine*, *methionine)***			
1.4.1.8	Valine dehydrogenase (NADP(+))	2	1
2.6.1.42	Branched-chain-amino-acid transaminase	2	4
1.2.4.4	3-methyl-2-oxobutanoate dehydrogenase	3	1
2.3.1.168	Dihydrolipoyllysine-residue (2-methylpropanoyl) transferase	2	0
1.3.99.3	Acyl-CoA dehydrogenase	3	1
1.3.99.12	2-methylacyl-CoA dehydrogenase	1	1
1.3.8.1	Butyryl-CoA dehydrogenase	2	1
4.2.1.17	Enoyl-CoA hydratase	2	2
3.1.2.4	3-hydroxyisobutyryl-CoA hydrolase	1	
1.1.1.35	3-hydroxyacyl-CoA dehydrogenase	3	1
2.3.1.16	Acetyl-CoA C-acyltransferase	3	2
1.1.1.31	3-hydroxyisobutyrate dehydrogenase	2	2
1.2.1.27	Methylmalonate-semialdehyde dehydrogenase (acylating)	2	1
6.4.1.3	Propionyl-CoA carboxylase	4	1
5.1.99.1	Methylmalonyl-CoA epimerase	0	0
5.4.99.2	Methylmalonyl-CoA mutase	0	1
2.5.1.6	Methionine adenosyltransferase	1	1
2.1.1.37	DNA (cytosine-5-)-methyltransferase	1	1
3.3.1.1	Adenosylhomocysteinase	2	1
4.2.1.22	Cystathionine beta-synthase	1	1
4.4.1.1	Cystathionine gamma-lyase	2	1
***Acetyl-CoA*, *acetoacetyl-CoA and acetoacetate degradation family (tryptophan*,*leucine*, *threonine*, *isoleucine*, *lysine*, *phenylalanine*, *tyrosine)***			
1.3.8.4	Isovaleryl-CoA dehydrogenase	2	1
6.4.1.4	Methylcrotonoyl-CoA carboxylase	1	1
4.2.1.18	Methylglutaconyl-CoA hydratase	1	1
4.1.3.4	Hydroxymethylglutaryl-CoA lyase	1	
1.2.1.10	Acetaldehyde dehydrogenase (acetylating)	1	1
2.3.1.9	Acetyl-CoA C-acetyltransferase	1	2
2.8.3.5	3-oxoacid CoA-transferase	2	3
2.3.3.10	Hydroxymethylglutaryl-CoA synthase	3	1
1.5.1.8	Lysine-ketoglutarate reductase	1	0
1.5.1.9	Saccharopine dehydrogenase		1
1.2.1.31	L-aminoadipate-semialdehyde dehydrogenase	1	1
2.6.1.39	2-aminoadipate transaminase	1	1
2.3.1.61	Dihydrolipoyllysine-residue succinyltransferase	1	2
1.3.99.7	Glutaryl-CoA dehydrogenase	1	1
1.2.4.2	Oxoglutarate dehydrogenase (succinyl-transferring)	8	1
**Total**		**115**	**90**

^a^The genes encoding multifunctional enzymes were shown in one cell.

### Molecular cloning, multiple sequence alignment and phylogenetic analysis

Out of those genes and ESTs, a putative *lkr/sdh* was identified and the sequence was confirmed by reverse transcription polymerase chain reaction (RT-PCR) using primers listed in Table 2. The 5'- and 3'-RACE fragments of *lkr/sdh* were amplified according to the manual of SMARTer RACE cDNA amplification kit (Takara Bio., Dalian, China), using antisense and sense gene-specific primers and universal primers. After obtaining the full-length cDNA, a pair of primers (Table 2) was designed to verify the complete open reading frame (ORF). The confirmed sequence (*Nllkr/sdh*) was submitted to GenBank (KJ958908).

**Table 2:** Primers used for RT-PCR, RACE, dsRNA synthesis and qRT-PCR.

Fragment name	Amplicon size (bp)	Forward sequence (5′-3′)	Reverse sequence (5′-3′)
**RT-PCR**			
*Nl*LKR/SDH	375	ACGCTACCCGATGCTAAA	TACGGAAGGATTGAAGTTGT
**RACE**			
5′ RACE		CCACTCCCACGCAATCACTCAC	
3′ RACE			AGCATCCGATACGCCGAACTCT
**ORF verification**			
*Nl*LKR/SDH	3110	AGTGAAGTAGCCTAGATGTG	AACTTTATTGAGAAATGGG
**dsRNA synthesis**			
dse*gfp*	460	CACAAGTTCAGCGTGTCCG	GTTCACCTTGATGCCGTTCT
dsLKR/SDH	598	CCACTCCCACGCAATCACT	GCATAGACCACCTGTTAGCCAT
**qPCR**			
qLKR/SDH	185	AGCTTCAAAGGACCAGGTGT	GCTATGATTGCTGCTTCTACG
*RPS15*	150	TAAAAATGGCAGACGAAGAGCCCAA	TTCCACGGTTGAAACGTCTGCG
*TUB*	174	ACTCGTTCGGAGGAGGCACC	GTTCCAGGGTGGTGTGGGTGGT

LKR/SDH-like proteins from twenty-two insect species were selected and aligned with the *Nl*LKR/SDH using ClustalW v. 2.1 [27]. The alignments were used to construct the maximum-likelihood (ML) trees using RAxML v.8 [28] to select the best-fitting model (LG+I+γ, with empirical frequencies) after estimation by ProtTest [29]. The reliability of ML tree topology was evaluated by bootstrapping a sample of 1,000 replicates. The LKR/SDH-like protein from *Ixodes scapularis* was added as an outgroup.

### dsRNA synthesis and bioassays

DNA samples for *Nllkr/sdh* dsRNA production and enhanced green fluorescent protein (*egfp*, control) were synthesized by PCR, using a 598 bp *Nllkr/sdh* and a 414 bp *egfp* fragment, and gene-specific primers (Table 2) incorporating the T7 RNA polymerase promoter sequence (5′-taatacgactcactataggg-3′). PCR products were purified using the Wizard SV Gel and PCR Clean-Up System (Promega, Madison, WI, USA) before used to synthesize dsRNAs with the T7 Ribomax Express RNAi System (Promega). The synthesized dsRNAs were respectively isopropanol-precipitated, resuspended in nuclease-free water, quantified by a spectrophotometry (NanoDrop 1000, Thermo Fisher Scientific, USA) at 260 nm, and kept at -80°C until use.

For bioassays, a previously reported dietary dsRNA-delivering procedure [30,31] was used with glass cylinders (12 cm in length and 2.8 cm in internal diameter) as feeding chambers using a chemically defined diet D-97 [32]. The bioassay had three treatments including a non-dsRNA diet (blank control), the diet containing ds*egfp* at the concentration of 0.50 mg/mL (negative control), and the diet containing ds*Nllkr/sdh* at the concentration of 0.50 mg/mL. Twenty *Ni*. *lugens* nymphs (three days after hatching) were carefully transferred into each feeding chamber of the different diet treatments. All treatments were replicated 15 times (15 chambers) with a total of 300 nymphs in each treatment (6 replicates for bioassays and 9 replicates for mRNA level evaluations). The experiments lasted for the whole nymphal stage. Mortality was recorded daily. Nymphal development duration and fresh weight of surviving adults were recorded.

### Quantitative real-time PCR (qRT-PCR)

Total RNA samples were prepared from whole bodies of the first- through fifth-instar (I1, I2, I3, I4 and I5) nymphs and adults, and from ventral ganglion (VG), thorax muscles (TM), epidermis (EP), fat body (FB), gut (GU) and ovary (OV) of normal adults or the adult survivors of the dsRNA bioassay using the SV Total RNA Isolation System Kit (Promega). mRNA abundance of the *Nllkr/sdh* in each sample was estimated by qRT-PCR (primers were listed in Table 1), using *ribosomal protein S15e* (*rps15*) and *alpha 2-tubulin* (*tub*) as internal control genes and the corresponding primer pairs reported recently [33]. All experiments were repeated in technical triplicate. Data were analyzed by the 2^-ΔΔCT^ method [34], using the geometric mean of *rps15* and *tub* for normalization according to the strategy described previously [34,35].

### Data analysis

The data were given as means ± SE, and were analyzed by one-way ANOVA followed by the Tukey-Kramer test, using SPSS for Windows (SPSS, Chicago, IL, USA).

## Results

### Identification of amino acid biosynthesis genes

According to *Ni*. *lugens* genome that was released recently, 115 amino acid degradation-related genes were obtained by manual annotations (Table 1, S1 Table). These genes encoded for 67 enzymes that were involved in metabolic degradation of amino acids (S1 Table). All these 115 genes had transcriptional evidence with 149 transcripts (S2 Table).

Ninety amino acid degradation-related genes were identified from YLS genome with manual annotations (Table 1). These genes shared the highest identities with that of *Metarhizium* spp, and encoded for 69 enzymes (S1 Table). All 90 genes had transcriptional evidence with 250 transcripts (S2 Table).

### Construction of amino acids degradation pathways

**Fig 1:**
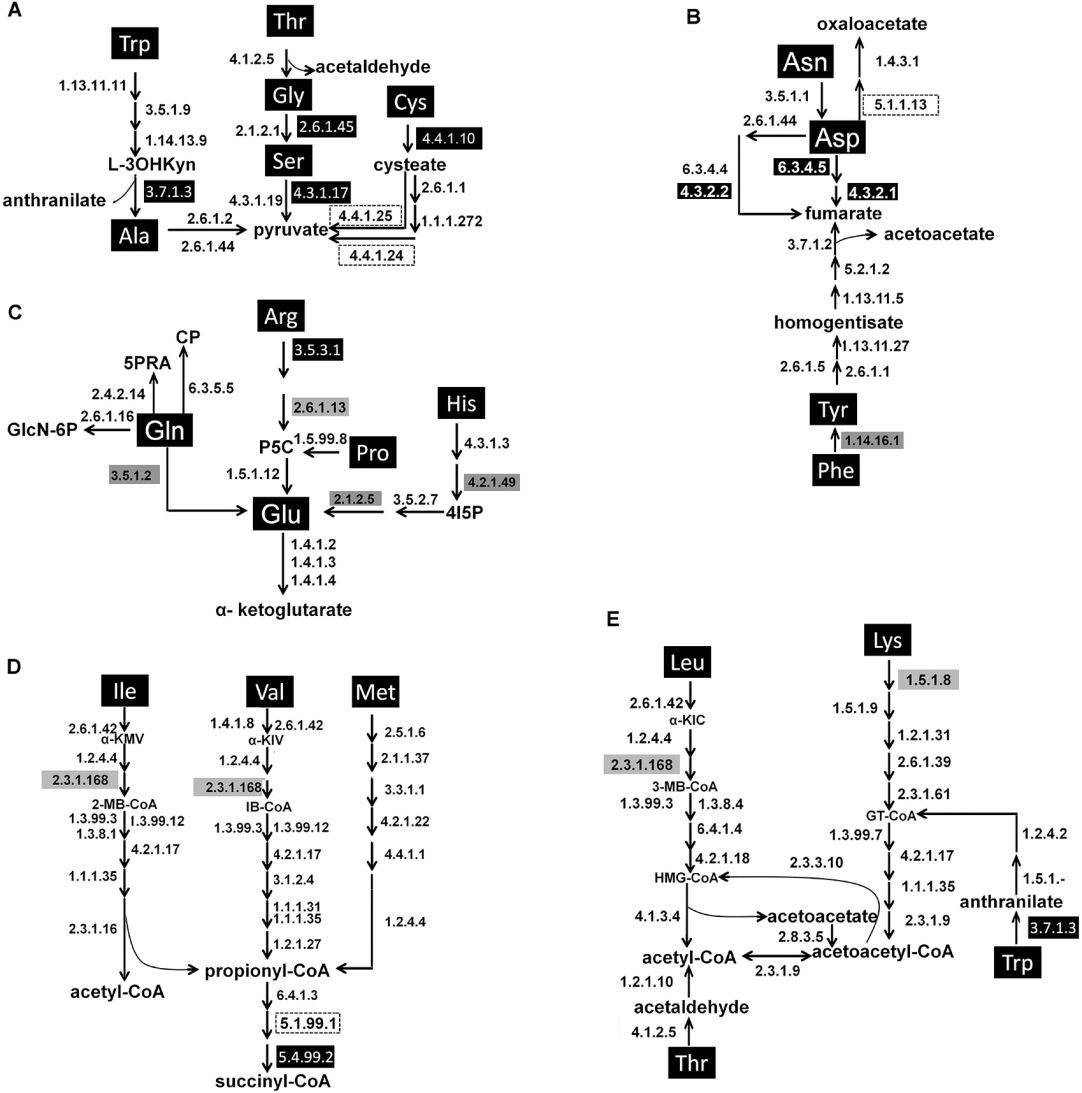
Proposed major degradation pathways of amino acids in *Nilaparvata lugens*. A) Pyruvate pathway; B) Oxaloacetate and fumarate pathway; C) α-Ketoglutarate pathway; D) Succinyl-CoAand acetyl-CoA pathway; and E) Acetoacetate pathway. The black arrows indicate reactions supported by genome and/or transcriptome annotation data. The numbers represent EC numbers. See Table 1 for details of the enzymes. Enzymes encoded by YLS genome are marked with white numbers in black boxes; the enzymes encoded by *Ni*. *lugens* genome are denoted with black number in grey boxes; the enzymes are homologous to those from both *Ni*. *lugens* and YLS genomes are given with black numbers without boxes; and the enzymes not found in *Ni*. *lugens* and YLS genome and transcriptome data are marked with black numbers in dashed boxes.

A total of 205 genes coding 77 enzymes were identified as *Ni*. *lugens* and/or YLS origin. These genes were used to construct metabolic degradation pathways of 20 protein amino acids (Fig 1) based on the Kyoto Encyclopedia of Genes and Genomic pathways and amino acid degradation pathways in *Ac*. *pisum* [36]. Common amino acids are degraded by 20 different pathways. The carbon skeletons converge to seven metabolic intermediates, i.e., pyruvate (Fig 1A), oxaloacetate and fumarate (Fig 1B), α-ketoglutarate (Fig 1C), acetoacetate, succinyl-CoA, and acetyl-CoA (Fig 1D and 1E).

**Fig 2:**
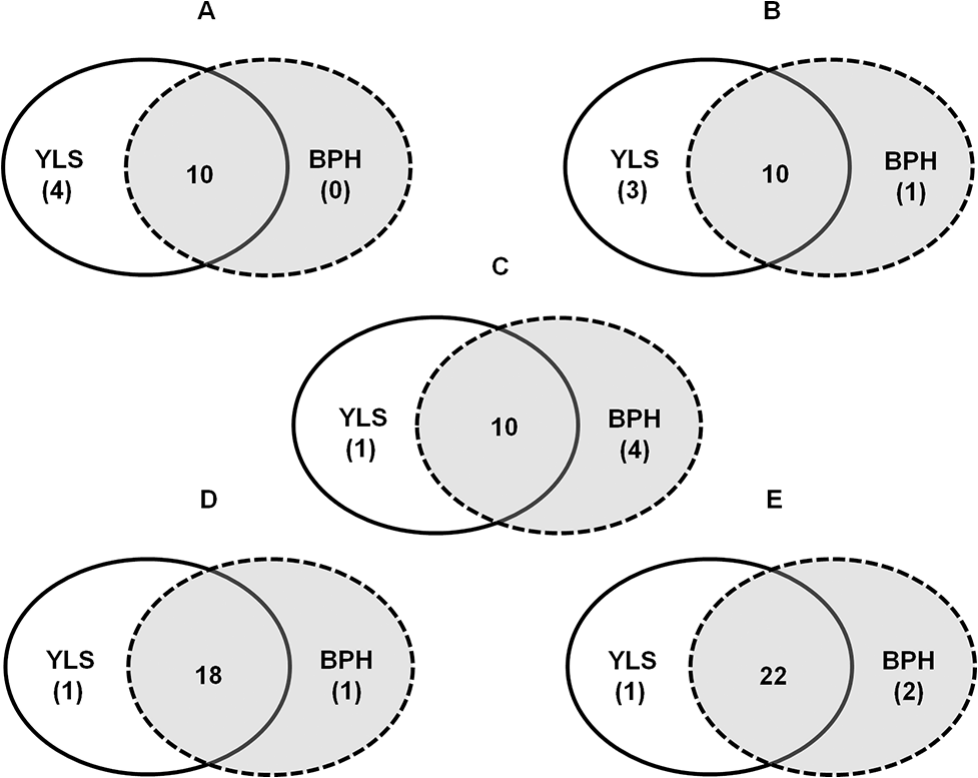
The number of enzymes from YLS and *Ni*. *lugens* (BPH) genome, respectively, involved in amino acid degradation. A) Pyruvate pathway; B) Oxaloacetate and fumarate pathway; C) α-Ketoglutarate pathway; D) Succinyl-CoAand acetyl-CoA pathway; and E) Acetoacetate pathway. Numbers in parentheses represent the number of enzymes from BPH or YLS, respectively. Numbers in the overlapping sections denote the number of enzymes originated from both BPH and YLS.

In the pyruvate pathway, *Ni*. *lugens* may independently degrade Ala, Thr, Gly and Ser, whereas YLS may independently break down Ala, Thr, Gly, Ser, Trp and Cys with two missing enzymes for the last step of Cys degradation (Figs 1A and 2A). In the oxaloacetate and fumarate pathway, both *Ni*. *lugens* and YLS have the complete set for the degradations of Asn, Asp, Tyr and Phe, except for the first step of the degradation of Phe (phenylalanine 4-monooxygenase, EC 1.14.16.1) and a missing enzyme for the first step of Asp degradation (aspartate racemase, EC 5.1.1.13) (Figs 1B and 2B). In the α-ketoglutarate pathway, *Ni*. *lugens* possesses a complete set of enzymes for the degradations of Gln, Glu, Pro and His, and Arg except for the first step (arginase, EC 3.5.3.1). YLS may only independently degrade Gln, Glu and Pro (Figs 1C and 2C). In the succinyl-CoA pathway, *Ni*. *lugens* has a complete set of enzymes for degradations of Ile, Val and Met, except for the enzymes of the last two steps (a missing enzyme Methylmalonyl-CoA epimerase, EC 5.1.99.1; methylmalonyl-CoA mutase, EC 5.4.99.2 of YLS). In contrast, YLS may not independently degrade any of Ile, Val and Met, due to lack of dihydrolipoyllysine-residue (EC 2.3.1.168) and/or EC 5.1.99.1 (Figs 1D and 2D). In the acetyl-CoA pathway, *Ni*. *lugens* has the complete enzyme set for the degradations of Leu, Thr, Lys and Trp, except for the enzyme of the first step (kynureninase, EC 3.7.1.3), and YLS may independently breakdown Thr and Trp.

### Molecular cloning and phylogenetic analysis of *Nllkr/sdh*

**Fig 3:**
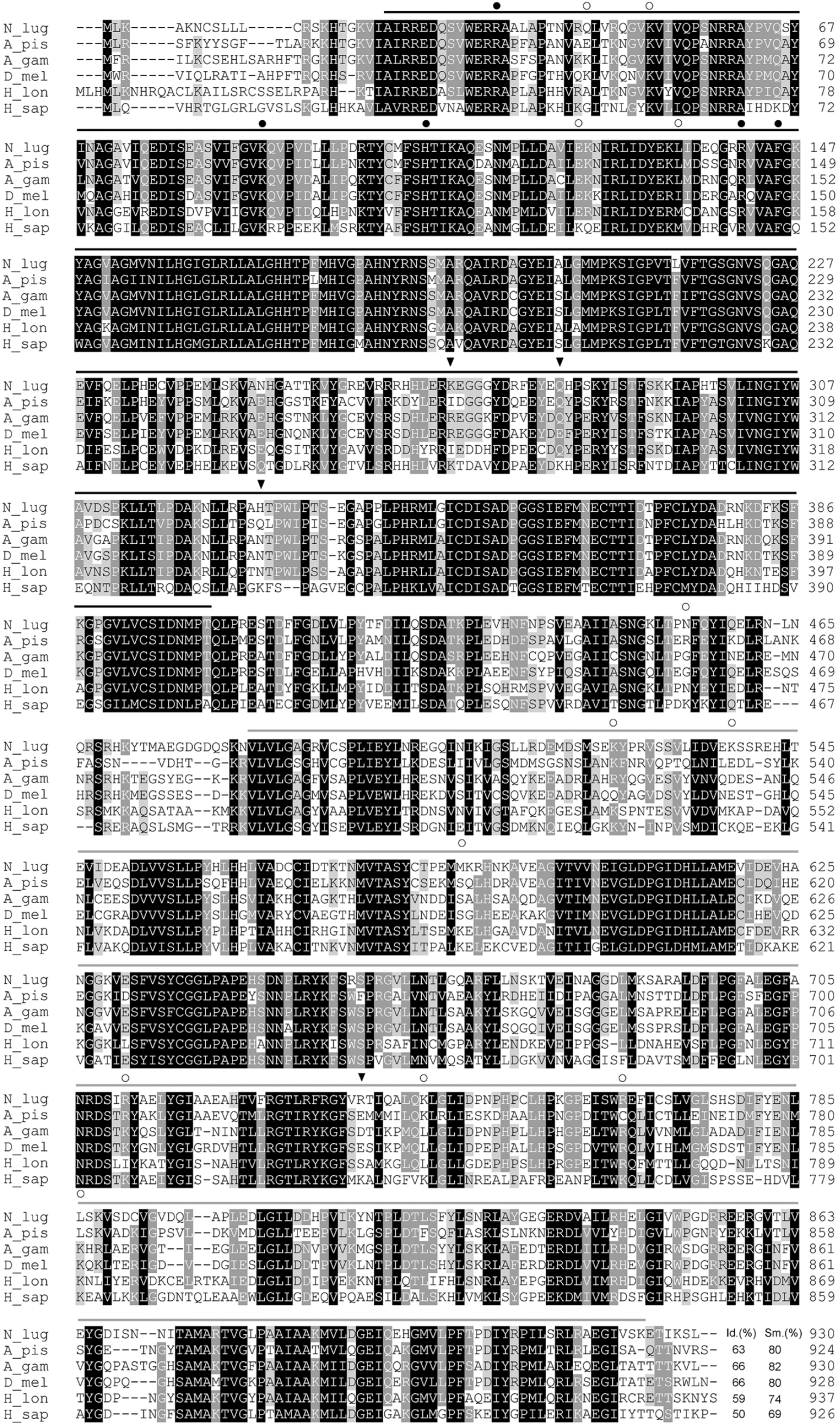
Sequence alignment of LKR/SDH. LKR/SDH originates from *Ni*. *lugens* (N_lug), *Acyrthosiphon pisum* (A_pis, ACYPI004937), *Anopheles gambiae* (A_gam, AGAP008632), *Drosophila melanogaster* (D_mel, FBpp0079118), *Haemaphysalis longicornis* (H_lon, BAI44335) and *Homo sapiens* (H_sap, CAA07619), respectively. Lysine α-ketoglutarate reductase and saccharopinee hydrogenase domains are marked with black line and gray line, respectively. The putative active sites, N6-acetyllysine sites and N6-succinyllysine sites are marked with full-filled cycles, empty cycles and triangles, respectively. Amino acids with 100%, >80%, and >60% conservation are shaded in black, dark grey and light grey, respectively. Gaps have been introduced to permit alignment.

Focusing on the degradation pathway of Lys, a gene showing significant homology to the bifunctional enzyme LKR/SDH was identified in the *Ni*. *lugens* genome and transcriptome. The full-length cDNA encoding the putative LKR/SDH consisted of 3,314 bp (*Nllkr/sdh*). The lengths of the 5′- and 3′-untranslated regions (UTRs) are 239 and 285 bp, respectively. The 3′-UTR ends with a 39 bp poly (A) tail begins at 21 bp downstream from AATAAA, the eukaryotic consensus polyadenylation signal. The ORF is 2,793 bp and encodes 930 amino acids with a predicted molecular mass of 103.2 kDa and a theoretical isoelectric point of 6.71 (Fig 3).

Domain structure analysis with the SMART program revealed that *Nl*LKR/SDH is a putative bifunctional enzyme with three distinct regions: an N terminal domain similar to LKR, a C-terminal domain similar to SDH, and an interposed short region connecting both domains. The LKR domain has five active sites, four N6-acetyllysine sites, and three N6-succinyllysine sites. Furthermore, seven N6-acetyllysine sites and one N6-succinyllysine site are located in the SDH domain. In addition, one N6-acetyllysine site is located in the interposed short region (Fig 3).

The *Nl*LKR/SDH shares the greatest similarity (86%) with LKR/SDH-like protein from *Rh*. *prolixus*. Similarly, it has 81% similarity with that from *Ac*. *pisum*, *Tr*. *castaneum* and *An*. *gambiae*, 80% similarity with that from *Bombus impatiens*, *Bo*. *terrestris*, *Ap*. *florae*, *Culex quinquefasciatus* and *Aedes aegypti*, 79%-71% similarity with *Ap*. *mellifera*, *Dr*. *melanogaster*, *Nasonia vitripennis*, *Atta cephalotes*, *Bo*. *mori*, *Pediculus humanus*, *Heliconius melpomene* and *Danaus plexippus*. Moreover, it shows 69%-46% similarity with those from *Plutella xylostella*, *Acromyrmex echinatior*, *Camponotus floridanus*, *Harpegnathos saltator* and *Ix*. *scapularis*.

**Fig 4:**
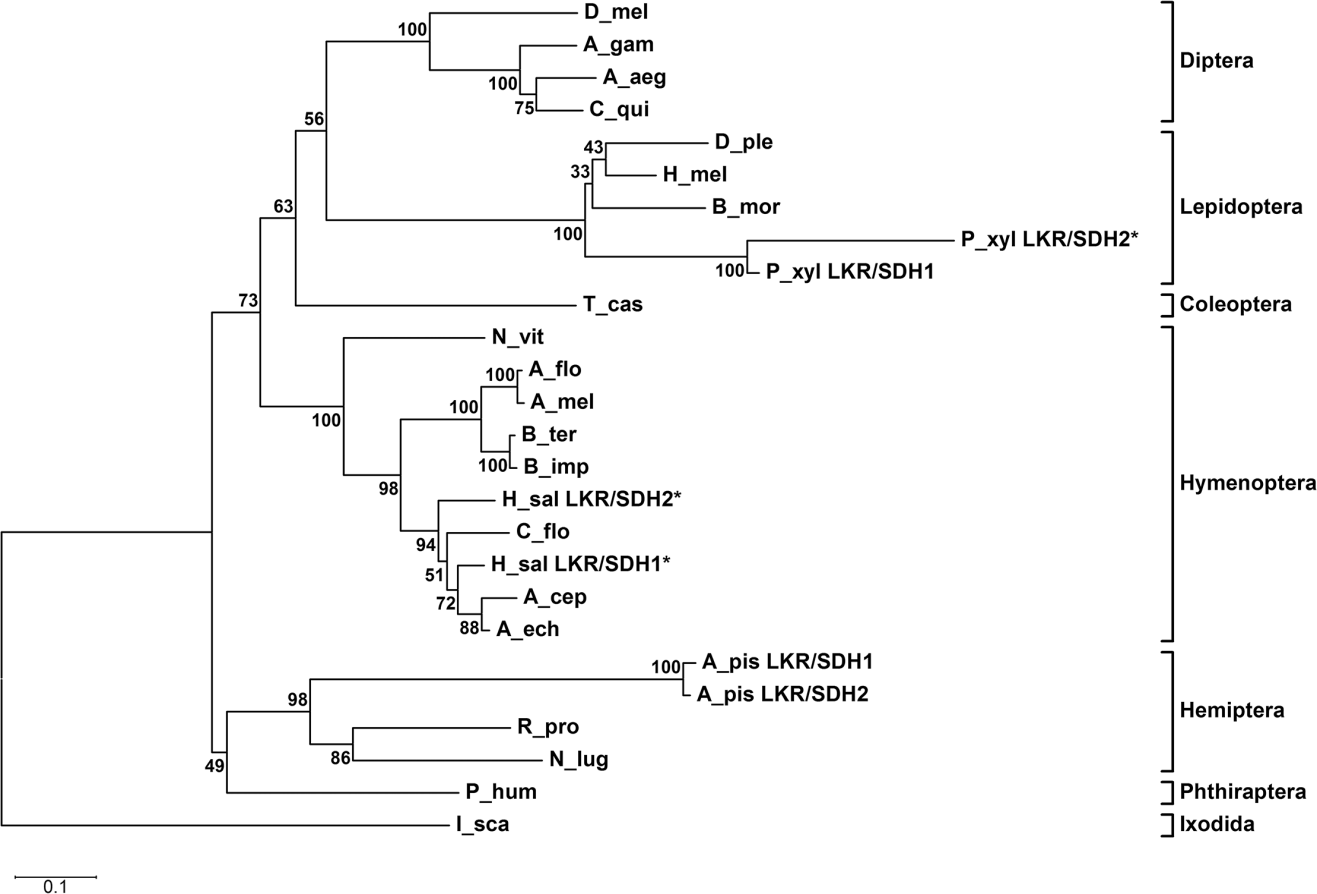
Phylogenic analysis of insect LKR/SDH. A rooted phylogenetic tree constructed by the maximum-likelihood method (the best-fitting model, LG+I+γ, with empirical frequency) based on the protein sequence alignments. The LKR/SDH-like sequences originated from *Ni*. *lugens* (N_lug), *Rhodnius prolixus* (R_pro, RPTMP07240), *Acyrthosiphon pisum* (A_pis LKR/SDH1, ACYPI004937; A_pis LKR/SDH2, ACYPI065217), *Pediculus humanus* (P_hum, PHUM016080), *Tribolium castaneum* (T_cas, TC002311), *Nasonia vitripennis* (N_vit, Nasvi2EG008769), *Acromyrmex echinatior* (A_ech, AECH18409), *Atta cephalotes* (A_cep, ACEP10511), *Camponotus floridanus* (C_flo, CFLO20285), *Harpegnathos saltator* (H_sal LKR/SDH1, HSAL12725; H_sal LKR/SDH2, HSAL12725), *Bombus impatiens* (B_imp, XP_003487153), *Bombus terrestris* (B_ter, XP_003398633), *Apis mellifera* (A_mel, GB47970), *Apis florea* (A_flo, XP_003692916), *Aedes aegypti* (A_aeg, AAEL014734), *Anopheles gambiae* (A_gam, AGAP008632), *Culex quinquefasciatus* (C_qui, CPIJ000416), *Drosophila melanogaster* (D_mel, FBgn0025687), *Bombyx mori* (B_mor, BGIBMGA010338), *Heliconius melpomene* (H_mel, HMEL016438), *Plutella xylostella* (P_xyl LKR/SDH1, Px002140; P_xyl LKR/SDH2, Px002187) and *Danaus plexippus* (D_ple, DPOGS208487).The LKR/SDH from *Ixodes scapularis* (I_sca, ISCW008489) was added as an outgroup. The percentiles of bootstrap values (1,000 replicates) are indicated. The scale bar represents the amino acid divergence. The pseudogenes are marked with asterisk.

Based on the amino acid sequences of LKR/SDH-like proteins from twenty-three species, a phylogenetic tree was constructed to evaluate the evolutionary relationships (Fig 4). The phylogenetic tree showed that the LKR/SDH-like proteins formed an Ixodida clade, a Phthiraptera clade, a Hemiptera clade, a Hymenoptera clade, a Lepidoptera clade, a Coleoptera clade and a Diptera clade. As expected, *Nl*LKR/SDH belongs to the Hemiptera clade (Fig 4).

### Expression patterns of *Nllkr/sdh*

**Fig 5:**
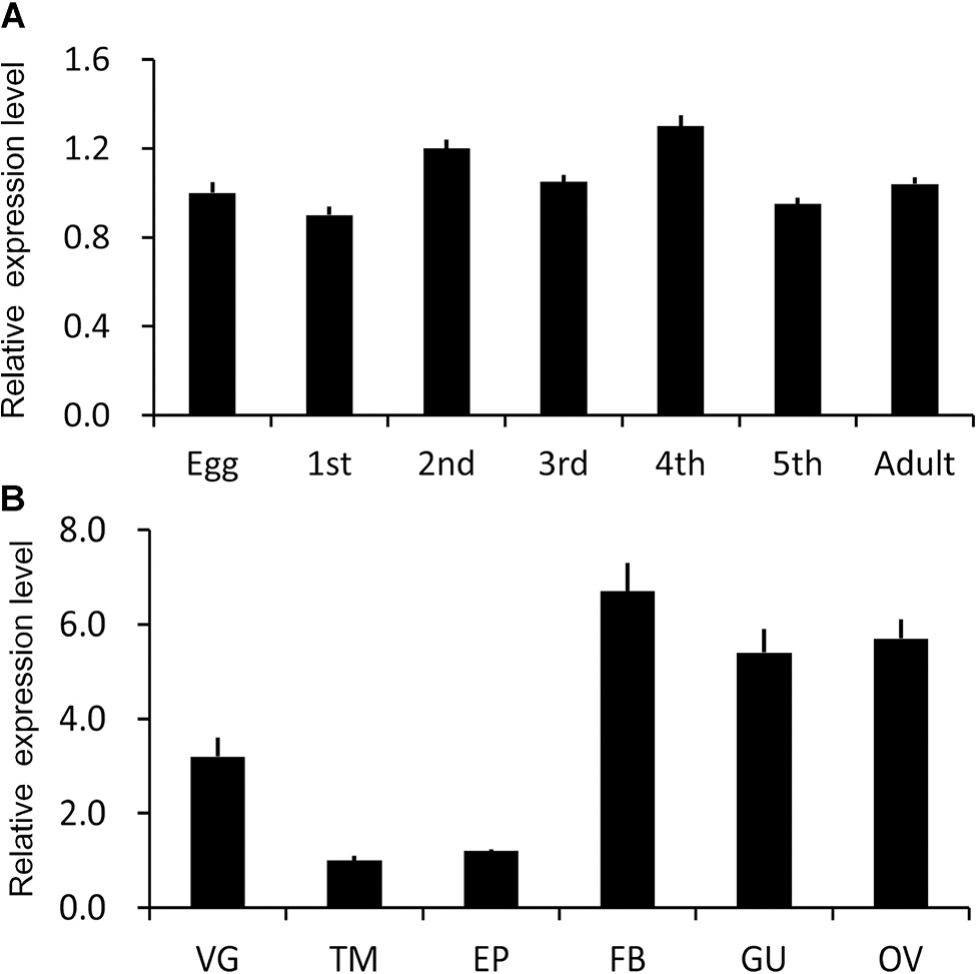
Temporal (A) and spatial (B) expression patterns of the putative *Nllkr/sdh*. cDNA templates were derived from eggs, first-, second-, third- and fourth-instar nymphs (1st, 2nd, 3rd, 4th, 5th), and adults, or from ventral ganglion (VG), thorax muscles (TM), epidermis (EP), fat body (FB), gut (GU) and ovary (OV) of adults. For each sample, 3 independent pools of 5–10 individuals were measured in technical triplicate using qRT-PCR. The values are calculated using the 2^-ΔΔCT^ method. The columns represent averages with vertical bars indicating SE.

The temporal transcript profile of *Nllkr/sdh* in nymphs and adults was analyzed using qRT-PCR. *Nllkr/sdh* was widely expressed in the eggs, first- through fifth-instar nymphs, and adults. The expression levels varied little among different development stages (Fig 5A).

The spatial mRNA level of *Nllkr/sdh* was measured with sexually mature adult females. *Nllkr/sdh* was highly expressed in the fat body, gut and ovary, moderately expressed in the ventral ganglion, and least expressed in epidermis and thorax muscles (Fig 5B).

### Effect of dsRNA on the expression of *Nllkr/sdh* mRNA level and planthopper development

**Fig 6:**
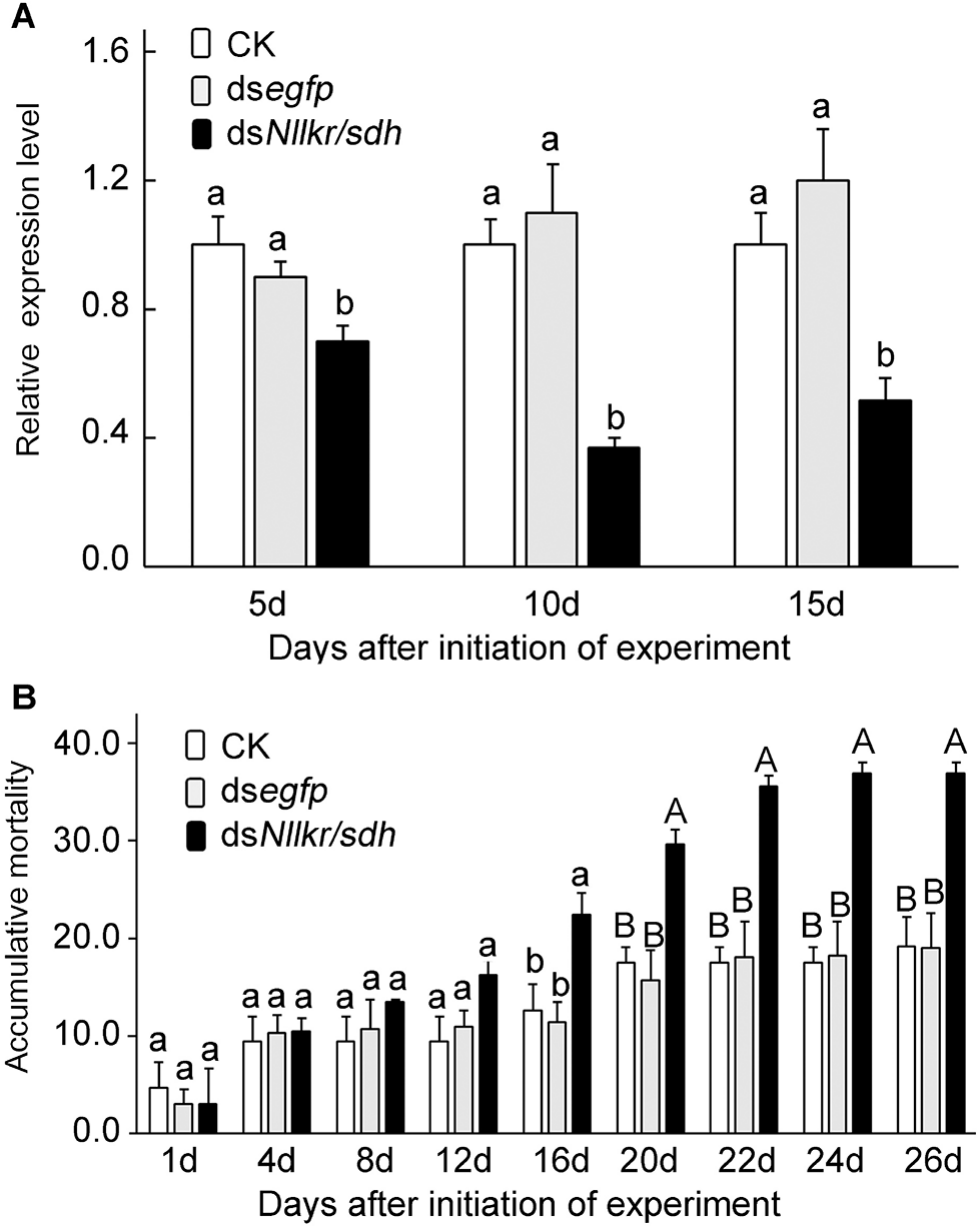
Ingestion of ds*Nllkr/sdh* on the mRNA level of *Nllkr/sdh* (A) and the survival (B) of the planthoppers. For the mRNA level, three biological replicates were conducted, and the mean ± SD (n = 3) was calculated to measure the relative transcript levels using the 2^−ΔΔCT^ method. For both the mRNA level and accumulative mortality, the columns represent averages with vertical lines indicating SE. Columns that do not share the same letter are significantly different at P value of 0.05.

After exposed to ds*Nllkr/sdh* for 5, 10 and 15 days, *Nllkr/sdh* mRNA abundance in surviving nymphs significantly decreased by 30.5%, 62.2% and 50.0%, respectively, compared with the blank control. In contrast, the *Nllkr/sdh* mRNA level in ds*egfp-*exposed nymphs was not significantly different from that of the controls (Fig 6A).

Continuous ingestion of ds*Nllkr/sdh* reduced survival of the planthoppers, compared with the blank control and the ds*egfp-*exposed nymphs. ANOVA analysis revealed that significant difference was observed 16 days after the initiation of ds*Nllkr/sdh* exposure and beyond. More hoppers died as the exposure duration increased (Fig 6B).

**Fig 7:**
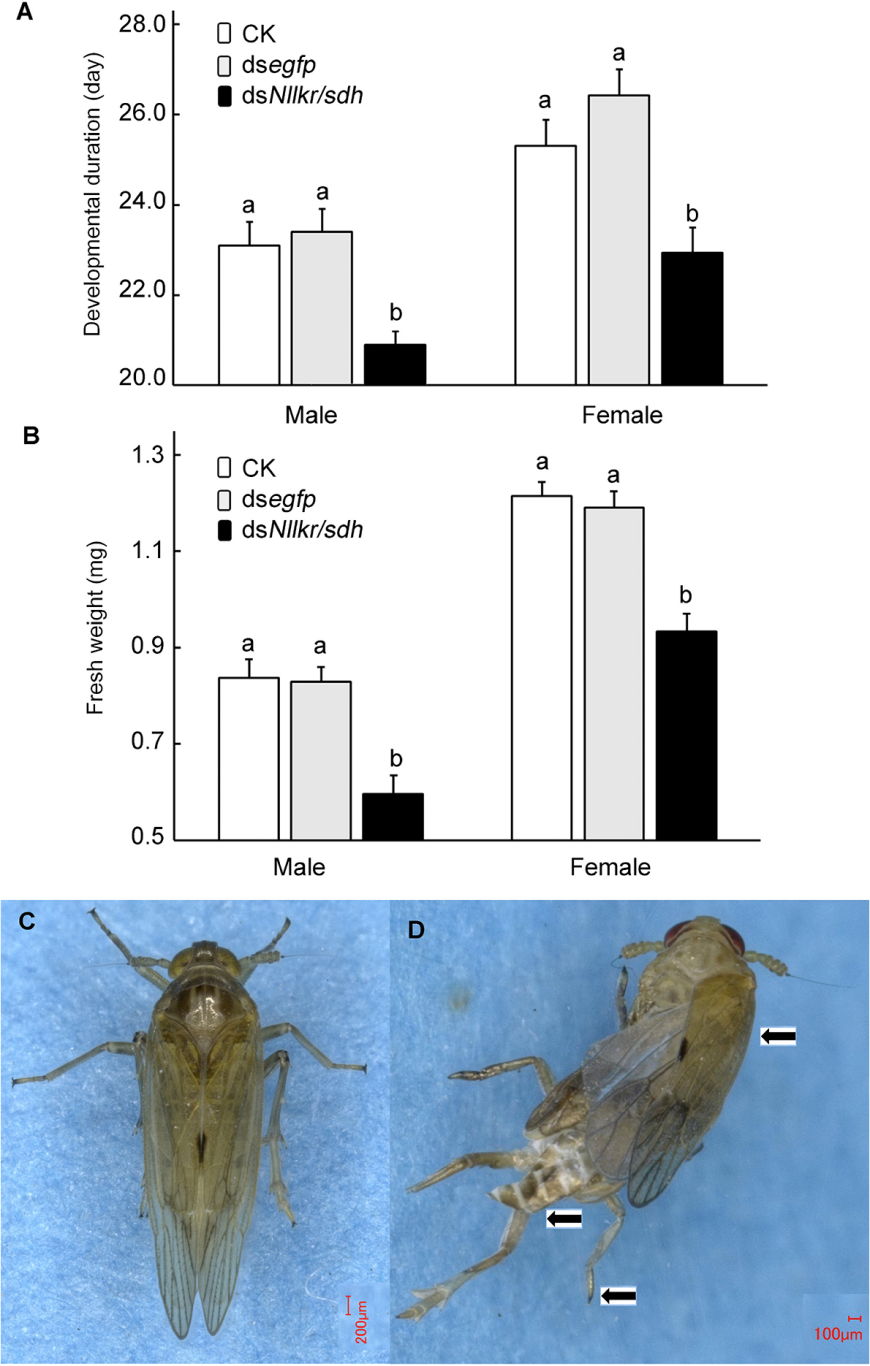
Ingestion of ds*Nllkr/sdh* negatively affects the development of planthoppers. Dietary introduction of ds*Nllkr/sdh* shortens the development duration (A), reduces the fresh weight (B), and causes abnormal defects. Two apparent phenotypic defects (D) observed in the resulting adults (C): wings were shortened and thickened, cuticle was semi-transparent, and old nymphal cuticles on the tips of legs and abdomens were not shed off (marked with arrows). The columns represent averages with vertical lines indicating SE. Columns that do not share the same letter are significantly different at P value of 0.05.

Male survivors ingesting normal, ds*egfp*-, or ds*Nllkr/sdh*-contained diet had average nymphal development durations of 25.0, 25.3 and 22.9 days, respectively. Similarly, female survivors having exposed to normal, ds*egfp*-, or ds*Nllkr/sdh*-contained diet had average nymphal development durations of 27.2, 28.3 and 24.8 days, respectively, with the former two being significantly longer than the latter (Fig 7A). The corresponding average fresh weights were 0.84, 0.83 and 0.59 mg, respectively, for males; and were 1.21, 1.18 and 0.92 mg, respectively, for females (Fig 7B).

Moreover, 65% of the surviving adults showed one or more of the three apparent phenotypic defects: (1) Wings were shortened and thickened, and were not well extended during adult ecdysis. (2) Cuticle was stretched and thinned, making it transparent as some of the internal organs were easily visible through the cuticle. And (3) Old nymphal exoskeletons remained on the tips of legs and abdomens and could not completely shed (Fig 7C and 7D).

## Discussion

*Ni*. *lugens* harbors several species of YLSs [15–17], mainly in abdominal fat bodies. According to *Ni*. *lugens* and YLS genome that were released recently [19], we identified 205 genes encoding 77 enzymes that are involved in the metabolic degradation of amino acids. Degradation pathways for the 20 protein amino acids were manually constructed.

In the present paper, we provided lines of evidence that in *Ni*. *lugens* some enzymes are encoded by genes originated from YLSs and others are encoded by genes of the hoppers. Firstly, all identified genes were successfully mapped to *Ni*. *lugens* or YLS genome, respectively [19]. Secondly, each of *Ni*. *lugens* and YLS gene has transcriptional evidence with EST support, respectively. Thirdly, each *Ni*. *lugens* and YLS gene shared a high identity with that of *Rh*. *prolix* and *Metarhizium* spp, respectively. It is postulated that *Ni*. *lugens* can independently catabolize fourteen amino acids (Thr, Ala, Gly, Ser, Asp, Asn, Phe, Tyr, Glu, Gln, Pro, His, Leu and Lys). *Ni*. *lugens* and symbiont enzymes may work collaboratively to catalyze the degradation of Trp, Cys, Arg, Ilv, Val and Met.

High levels of Lys were toxic to plant and mammalian cells [5–7]. High-Lys or high-protein diets increased LKR and SDH activities in rat liver [37,38]. Also, LKR and SDH activities as well as LKR/SDH mRNA increased in mice receiving Lys or saccharopine injections or diets containing excessive Lys [7,39]. Furthermore, mRNA levels increased in the midgut, salivary gland and fat body of *Ha*. *longicornis* ticks injected with Lys or saccharopine [2]. A Lys catabolic pathway is an important route for balancing Lys levels. Thus, we cloned *Nllkr/sdh* in *Ni*. *lugens*. The *Nl*LKR/SDH protein is a putative bifunctional enzyme with three distinct regions. The phylogenetic analysis revealed that the LKR/SDH-like proteins from twenty-two insect species formed clades agreed with taxonomy. The *Nl*LKR/SDH belongs to the Hemiptera clade as expected. From insect genome data, one *lkr/sdh* gene was discovered in *Ae*. *aegypti*, *Ap*. *mellifera*, *An*. *gambiae*, *Bo*. *mori*, *Cu*. *quinquefasciatus*, *Da*. *plexippus*, *Dr*. *melanogaster*, *Pe*. *humanus*, and *Na*. *vitripennis*. Two *lkr/sdh*genes were detected in *Ha*. *saltator*, *Pl*. *xylostella* and *Ac*.*pisum*, indicating a gene duplication event within the genome. Out of those genes, one or both *lkr/sdh* is pseudogenes that result from premature stop codons in *Ha*. *saltator*, and *Pl*. *xylostella*, whereas two *lkr/sdh* paralogs are protein-coding genes in *Ac*. *pisum*. It appears that all insect *lkr/sdh*s have evolved from a common ancestral gene, and gene duplication or loss have occurred during the evolution. In addition, LKR/SDHs from several insects, including *Ac*. *pisum* are annotated as mitochondrial-like or simply mitochondrial proteins. In mammals, lysine is catabolized via mitochondria through the bifunctional enzyme alpha-aminoadipic semialdehyde synthase that is known as LKR/SDH [40,41]. It suggests that *Nl*LKR/SDH is encoded in the nucleus and is probably transported to the mitochondria to function.

*Nllkr/sdh* was widely expressed in the first- through fifth-instar nymphs and adults. The expression levels were similar among different development stages. Similarly, *Hllkr/sdh* was expressed in all developmental stages of *Ha*. *longicornis* [2]. This universal expression pattern suggests an important role of LKR/SDH in insect or tick development.

The present work provides three pieces of empirical evidence regarding the importance of LKR/DSH. Firstly, the ingestion of ds*Nllkr/sdh* successfully knocked down the target gene, and reduced the survivorship of the planthoppers. Similarly, all *Ha*. *longicornis* ticks injected with ds*Hllkr/sdh* died 25 days after engorgement due to irreversible pathological changes [2]. Moreover, the spatial distribution of *Nllkr/sdh* indicates its importance in the survival and development of *Ni*. *lugens* as *Nllkr/sdh* was highly expressed in vital organs such as the fat body and gut (important organs in insects), moderately expressed in the ventral ganglion, and least expressed in epidermis and thorax muscles. Similarly, *Hllkr/sdh* was highly expressed in the midgut, fat body and synganglion in unfed *Ha*. *longicornis* ticks [2]. LKR/SDH enzyme activities are important for Lys catabolism in the liver and kidney of mammals [3,42], contributing not only to the general nitrogen balance but also to the controlled conversion of Lys into ketone bodies [3,37,39,42]. The fat body in arthropods is an organ that stores energy, metabolizes hormones and other essential messenger molecules and detoxifies wastes or harmful compounds. Its functional value is comparable to that of the liver of vertebrates [43]. The high expression level of *lkr/sdh* in fat bodies indicates that the insect LKR/SDH may play similar roles to that in mammals. Furthermore, Lys is an important precursor for synthesis of Glu, the most significant excitatory neurotransmitter in mammalian as well as arthropod central nervous systems [44]. The widespread distribution of LKR/SDH in the central nervous systems suggests that Lys is an important precursor of Glu in mammals and arthropods.

Secondly, we found that knocking down of *Nllkr/sdh* shortened *Ni*. *lugens* nymphal development and decreased adult fresh weight. The *Dm*LKR/SDH in *Dr*. *melanogaster* is involved in ecdysone-mediated transcription. *Dm*LKR/SDH binds histone H3 and H4 and suppresses ecdysone-mediated transcription of cell death genes by inhibiting histone H3R17me2. In the absence of *Dm*LKR/SDH, histone methylation occurs prematurely and enhances hormone-regulated gene expression to affect the developmental timing. Homozygous deficiency of *Dmlkr/sdh* produces viable adult flies that exhibit significantly smaller wing size and reduced overall body weight [45]. Similar LKR/SDH deficiency phenotypes of *Ni*. *lugens* suggest that *Nl*LKR/SDH may also be involved in ecdysone-mediated transcriptions.

Finally, silencing *Nllkr/sdh* caused the three morphological defects associated with wings and cuticles, a sign of imbalanced osmotic pressure. In arthropods, the Malpighian tubules and guts play a major role in salt and water balance. The Malpighian tubules and rectum of insects form a physiological complex that serves as a functional kidney at the organism level. Urine is secreted in the Malpighian tubules, and transported to the rectum for selectively reabsorbing ions and water. This processes of secretion and reabsorption are responsible for maintaining osmotic balance between the intracellular and extracellular compartments [12]. In *Ha*. *longicornis*, ds*Hllkr/sdh* injection induced 2–4 times greater volume of hemolymph than the control groups. Pathomorphological examination showed that the midgut, Malpighian tubules and rectal sac were filled with a watery liquid. This high volume of hemolymph created a high hydraulic pressure and stretched the tick cuticle thin and transparent so that all the internal organs were easily visible through the cuticle. In addition, the Gene’s organ protruded with a hernia-like morphology is probably due to the high hydraulic pressure from the large volume of hemolymph. Lower amounts of guanine crystals were observed in the rectal sac and Malpighian tubules [2]. All those results indicate that LKR/SDH enzymes regulate osmotic stress. In plants, LKR level is significantly up-regulated in inflorescence tissues and developing seeds, and as a response to osmotic stress [46–49]. Other studies reported that LKR activity increases when the osmotic stress becomes high [48,49]. In rapeseed, the LKR/SDH gene is most responsive to osmotic stress [49,50]. Furthermore, LKR/SDH may play a role in egg production. A study with *Ha*. *longicornis* showed that *Hllkr/sdh* was highly expressed in the ovary. RNAi-mediated knockdown of *Hllkr/sdh* showed a clear influence on egg production and tick reproduction [2]. In this study, our results revealed that *Nllkr/sdh* was highly expressed in the ovary. The potential effect of *Nllkr/sdh* knock-down on reproduction of *Ni*. *lugens* is currently under evaluation in our laboratory. The physiological roles of other amino acid catabolism genes also will be delineated through RNAi knockdown in future.

## Supporting Information

S1 Table The genomic positions of identified *Ni*. *lugens* and YLS genes that invloved amino acid degradation. 1A: Pyruvate degradation family; 1B: Oxaloacetate and fumarate degradation family; 1C: α-ketoglutarate degradation family; 1D: Succinyl-CoA degradation family; 1E, Acetyl-CoA, acetoacetyl-CoA and acetoacetate degradation family.
(XLS)

S2 Table The number of ESTs that were mapped to identified *Ni*. *lugens* and YLS genes, respectively. (XLS)
